# Hospital incident command groups’ performance during major incident simulations: a prospective observational study

**DOI:** 10.1186/s13049-020-00763-4

**Published:** 2020-07-29

**Authors:** Jason P. Murphy, Lisa Kurland, Monica Rådestad, Anders Rüter

**Affiliations:** 1 Department of Clinical Science and Education, Karolinska Insititutet, Stockholm, Sweden; 2 Sophiahemmet University, PO Box 5605, SE-11486 Stockholm, Sweden; 3 Department of Medical Sciences, Örebro University, Örebro, Sweden; 4 Department of Neurobiology and Society, Karolinska Institutet, Stockholm, Sweden

**Keywords:** Hospital disaster preparedness, Hospital incident command group, Performance indicators, Simulation exercises, Major incident, Hospital management, Decision-making

## Abstract

**Background:**

Hospital incident command groups’ (HICG) performance may have a profound impact on hospital response to major incidents. Previous research has assessed hospital incident command group capacity as opposed to performance and factors associated to performance. The objective was to assess associations between decision-making and staff procedure skills of the hospital incident command group.

**Methods:**

This was a prospective observational study using performance indicators to assess hospital incident command groups’ decision-making and performance. A total of six hospitals in Stockholm, Sweden, with their respective HICGs participated. Associations between decision-making skills and staff procedure skills during major incident simulations were assessed using measurable performance indicators.

**Results:**

Decision-making skills are correlated to staff procedure skills and overall HICG performance. Proactive decision-making skills had significantly lower means than reactive decision-making skills and are significantly correlated to staff procedure skills.

**Conclusion:**

There is a significant correlation between decision-making skills and staff procedural skills. Hospital incident command groups’ proactive decision-making abilities tended to be less developed than reactive decision-making abilities. These proactive decision-making skills may be a predictive factor for overall hospital incident command group performance. A lack of proactive decision-making ability may hamper efforts to mitigate the effects of a major incident.

## Background

Hospitals play vital roles during major incidents (MI) [1]. Previous studies have demonstrated that well-prepared hospitals may mitigate the impact of an MI as measured by morbidity and mortality [2–4]. A consensus concerning a standardized method for assessing hospital disaster preparedness is lacking despite directives stipulating the need for hospital disaster preparedness [5, 6]. Hospital response and performance is reliant on hospital management [7]. Hospital emergency contingency plans include descriptions the hospital incident command group (HICG). This group, responsible for coordinating medical care, personnel and allocation of resources consists of an incident commander and representatives from amongst others, logistics, the ED, surgical units, the ICU, security, and communications [7]. While addressing aspects of hospital management e.g. capacity, there are few studies focusing on HICGs’ performance [4, 7–9]. However, there is mounting evidence suggesting that performance of the HICG as opposed to capacity can and should be assessed [10–12].

### Measuring hospital incident command performance

Decisions and actions taken by the HICG during the initial phase of an incident are essential for managing resources during a major incident and may affect patient outcomes [13]. Successful management of limited resources is contingent on planning, training and timely responses concerning the mobilization of limited resources [14]. Of importance is the ability to mobilize resources to meet medical demands before all facts of an incident are known, relying on anticipatory or analytical abilities [3]. It has been demonstrated that the HICG’s ability to work in a structured fashion and its decision-making skills can be assessed by analyzing measurable indicators (Tables 1 and 2) [6, 10]. The Disaster Management Indicator (DiMI) instrument which is based on process modeling and constructed through consensus by the Swedish National Board of Health and Welfare [6] is to the authors knowledge the only tool measuring HICG performance.

**Table 1:** Scores, Median and Mean for decision-making skills

Performance Indicator (standard within x minutes)		Simulations						Median	Mean
		A	B	C	D	E	F		
1	Decision concerning hospital level of preparedness [3]	1	2	2	2	2	2	2	1,83
2	Initial guidelines for hospital response formulated [15]	2	2	2	1	2	2	2	1,67
3	First information to media [15]	1	1	2	2	2	0	1.5	1,5
4	Information concerning resources reported to the strategic level of management (25)	1	2	2	2	2	2	2	1,83
5	Medical officers appointed at emergency and surgical departments (30)	2	2	2	2	2	2	2	2
6*	Needs of ICU capacity estimated (45)	1	2	1	1	2	2	1.5	1,5
7	First information to hospital staff (60)	2	2	2	2	2	2	2	2
8*	Endurance of staff estimated (90)	1	1	1	1	2	2	1	1,3
9*	Shortage of own capacity estimated and reported (120)	2	1	1	1	2	2	1.5	1,5
10*	Influence on daily hospital activities estimated (120)	0	2	2	1	1	2	1.5	1,3
11*	Plan for patients with postponed appointments and operations formulated (180)	0	2	0	0	0	2	0.00	0,67
Total score		13	19	17	15	19	20		17.16

Indicator related to proactive decision-making indicated with (*)

**Table 2:** Scores, Median and Mean staff procedure skills

Performance indicator (standard within x minutes)		Simulation						Median	Mean
		A	B	C	D	E	F		
12	Functions to staff members assigned (direct)	2	2	2	2	2	2	2	2
13	Positioning in room in accordance to above (direct)	2	2	2	2	2	2	2	2
14	Designated telephone numbers (direct)	2	2	2	2	2	2	2	2
15	Arriving staff members introduced (1 min)	1	2	2	0	2	2	2	1,5
16	Equipment utilize (only if equipment is available)	2	2	2	2	2	2	2	2
17	Staff briefing (max 8 min in length)	2	2	1	2	1	2	1.5	1,5
18*	Content of staff briefing	1.75	2	2	1.75	2	2	2	1,92
19	Telephone discipline	0	1	1	0	1	2	1.25	1,08
20	Content of staff schedule	2	1	1	2	2	0.5	2	1,67
21	Summary: oral briefing	2	2	2	2	2	2	2	2
22	Summary: written	2	2	0	2	2	2	2	2
Total		18.75	19	17	15	19	20.5		19,7

* consists of sub indicators as described [6]

The DiMI allows for assessment of HICG performance by analyzing measurable indicators reflective of the operations of the HICG. The DiMI assesses whether actions relating to structures, process and decision-making were performed through two focal points; HICG’s decision-making ability and staff procedure skills which is the staff’s ability to work in a structured and organized way [6, 15]. A previous study providing a first analysis of associations between the two skill sets, identified a linear association between staff procedure and decision-making abilities and indicated that improved staff procedure skills would lead to improved decision-making skills [15]. DiMI decision-making indicators can be divided into two sub-groups of indicators, reactive and proactive decision-making. Reactive decision-making can be defined as intuitive, reflexive decisions based on previous experiences and knowledge while utilizing minimal cognitive resources [16, 17]. Conversely, proactive decision-making may be defined as anticipatory, time consuming, deliberate requiring analytical process and is more demanding of cognitive efforts [16, 17].

There are to our knowledge, few prospective observational studies focusing on the association between decision-making skills and staff procedure skills and no studies analyzing the association between proactive decision-making and staff procedure skills during a simulated major incident.

### Aim

The aim was to assess associations between decision-making skills and staff procedure skills of hospital incident command groups during major incident simulations using performance indicators as measured by DiMI.

## Method

This was a prospective observational study using performance indicators to assess hospital incident command groups’ decision-making and performance.

### Study setting

Six consecutive tabletop simulation exercises at six separate major hospitals (A-E. Tables 1 and 2) were conducted during the fall of 2016 in the region of Stockholm, Sweden during the period of October 2016 to December 2016. All six simulations planned and carried out by the regional hospital disaster preparedness coordinators in Stockholm, Sweden, were antagonistic scenarios based on prior terrorist incidents; five bomb blast scenarios and one active shooter scenario. The incident was presented as realistically as possible, i.e. the conditions for the participating hospitals were consistent with the real time personnel, resources and information. Information concerning the nature of the respective incidents was withheld from participants prior to the exercises, they were only informed of the date and approximate time. The extent of the simulations varied, i.e. some simulation exercises included other parts of the hospital, while others focused solely on the HICG. In both instances, the HICG had access to all units and representatives per emergency contingency plans, facilitating similar conditions for evaluation of the HICG.

The designated hospital incident command groups, which are activated in accordance to the hospital disaster management plans, were the study subjects.

### Data collection

Data collection was based on observation and included variables as required by the DiMI [6]. The DiMI consists of 22 measurable indicators divided into two groups of 11 indicators with 11 measuring decision-making skills and 11 measuring staff procedure skills. The observers (JM and AR) were present in the hospital incident command room throughout the entire duration of each simulation. Written documentation and logfiles from the HICGs were obtained after completion of the simulations in order to ensure accurate documentation.

Time standards for indicators were reached through expert consensus [18, 19]. The indicators reflecting decision-making skills consist of six reactive and five proactive decision-making indicators Table 1 [17]. Each indicator was scored on a scale from 0 to 2. A value of 0 indicates that the standard for the indicator was not completed. A value of 1 indicates that the standard for the indicator is partially completed or not completed within the predefined required time frame. A value of 2 indicates that the standard for the indicator was completed correctly and within the predefined required time frame.

### Data analysis

Data from all simulations was first imported to Microsoft Excel for Mac version 16.33 and analyzed using descriptive and inferential statistics.

Individual indicators were analyzed using ANOVA and DUNN post hoc analysis. Differences in means for decision-making and staff procedure skills were assessed using one-way ANOVA and Kruskal-Wallis Test. Pearson’s correlation was used to assess the association between decision-making and staff procedure skills. Due to the data being rank-order data as well as a lack of assumption concerning the distribution of data, a Spearman’s rho correlation coefficient was computed to measure the degree of association between the different groups of indicators, i.e. decision-making and staff procedure skills and subgroups of decision-making skills.

A ρ value < 0.05 was considered significant.

Data analysis was conducted using JASP version 0.9.2(JASP Team 2018) and Statistical Package for Social Sciences (SPSS) version 25 (IBM SPSS Statistics North Castle, New York, USA).

## Results

The duration of each simulation ranged from 2 h and 13 min to 6 h and 52 min. Medians and mean scores are presented. Mean scores are used with the aim of more accurately highlighting subtle, yet significant differences in performance. For instance, a mean score of 0.67 is closer to 1, than the median of 0.000, indicating that a task, was performed to a certain degree (0.67), as opposed to “not at all” that a median of 0 would indicate. The mean score for the decision-making indicators ranged from 0.67 to 2.0 while mean scores for staff procedure indicators ranged from 1.08 to 2.0. The sum of the mean scores for all six simulations concerning decision-making was 17.16 (Table 1) while the sum of the mean scores for staff procedure skills was 19.66 (Table 2).

Indicator related to proactive decision-making indicated with (*).

### Association of indicators

A one-way analysis of variance indicated a statistically significant differences between the decision-making skills and staff procedure (*p* = 0.036, d = 0.386) (Table 3).

**Table 3:** Post Hoc Comparisons – Decision-making and staff procedure means

		Mean Difference	SE	t	Cohen’s d	p _tukey_
1	2	−0.227	0.103	−2.215	−0.386	0.028
*Kruskal-Wallis Test*						
Factor	Statistic	df	p			
Role	4.398	1	0.036			

**Fig. 1:**
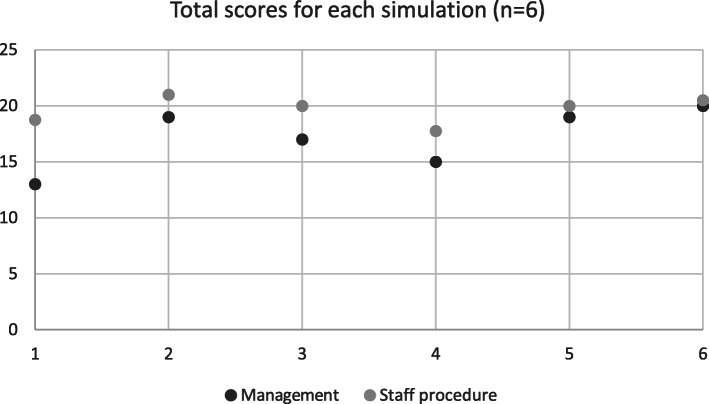
Correlation Decision-making and staff procedural skills r = 0.809, *p* = 0.051

The correlation between decision-making skills and staff procedure skills was r = 0.809, ρ = 0.51 (Fig. 1, Table 4).

**Table 4:** Spearman’s rho correlation of decision-making and staff procedure skills

	Staff procedure skills		ρ
Decision-making		.809	.051
Proactive decision-making		.947	.014
Reactive decision-making		.090	.86

*. Correlation is significant at the 0.05 level (2-tailed)

**Fig. 2:**
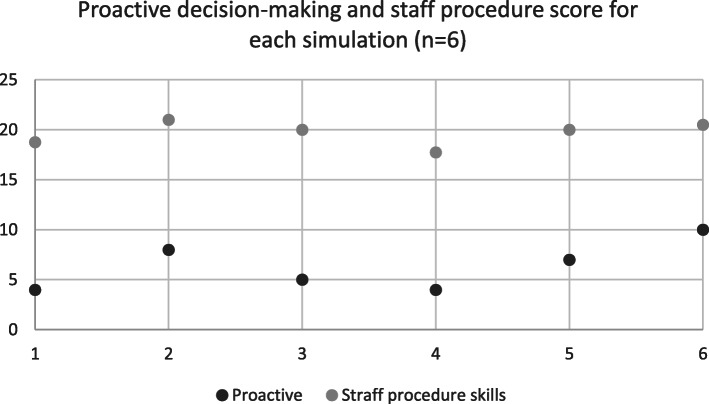
Correlation proactive decision-making and staff procedure r = 0,947 *p* = .014

Reactive skills had statistically significant higher means (1.5–2.0) than proactive skills which had lower means (0,80–1.60) (*p* = .046) (Table 5). While Spearman’s rho indicated no significant correlation between reactive indicators and staff procedure (r = 0,09 and *p* = .86) there was significant positive correlation between proactive indicators and staff procedure skills (r = 0,947 *p* = .014) (Fig. 2, Table 4).

**Table 5:** Reactive and proactive indicator means

	N	Minimum	Maximum	Mean	ρ
Reactive	6	1,66	2,00	1,83	.046
Proactive	5	,80	1,60	1,07

## Discussion

The current study identified a relationship between decision-making and staff procedure skills. In addition, this study identified a correlation between proactive decision-making and staff procedure skills. Proactive decision-making skills in particular may therefore have an impact on overall disaster performance of the HICG. Hospital incident command groups with lower scores for proactive decision-making skills, had statistically significantly lower performance scores. While a previous study suggested that improved staff procedure skills resulted in improved decision-making skills [15], to our knowledge, this is the first study to demonstrate an association between proactive decision-making and HICG disaster performance. Given the type of data and the nature of this study, it is not possible to state causation. However, based on these results, decision-making skills may provide the foundation needed for effective staff performance and overall hospital response. The positive correlation between decision-making and staff procedure skills identified in this study illustrate the need to further explore the possible causative relationships and motivate the need for further research.

The significant difference between reactive decision-making indicators and proactive decision-making indicators with respect to HICG performance measured by DiMI is noteworthy. Reactive decisions are typically made during the early stages of an incident e.g. decisions on the level of preparedness, were more often correctly executed within the predetermined time frames. Conversely, proactive decisions based on estimations, e.g. the delivery of information pertinent to staff stamina, or estimating influence on daily activities, were delayed or not made, consistent with a previous retrospective study assessing decision-making [11] . Of particular interest is the correlation between proactive decision-making indicators and staff procedure skills. The statistically lower means for proactive decision-making skills indicate that analytical skills may be an underdeveloped yet vital component as indicated by their correlation with staff procedure skills. The importance of analytical/anticipatory abilities is further illustrated by the lack of correlation between reactive decision-making and staff procedure skills. Previous research has demonstrated that experience is an important aspect for analytical ability [20, 21]. While not controlled for in this study, lower proactive abilities may be a result of a lack of experience or knowledge as reported in a 2007 study assessing proactive vs reactive decision-making in the clinical setting [21].

While this study reports acceptable levels of HICG disaster preparedness, the frequency of training required to maintain or improve preparedness is an important factor to consider.

This study also further demonstrated that measurable indicators may be an effective method for facilitating a structured evaluation of the hospital incident command group. Furthermore, this study suggests that the DiMI may facilitate HICG performance if implemented as a guide for the HICG. While the DiMI is an efficient method for evaluating HICG preparedness, the DiMI may also be compatible with other methods such as checklists, interviews or questionnaires.

In addition to factors such as training, repetition and effectivity, this study indicates the need to recognize and improve analytical skills. Furthermore, these findings may guide pedagogical construction of training and educational programs targeting these skills. This in turn, may enhance HICG’s disaster management.

### Limitations

The data was treated as interval data in order to make the results comparative with earlier studies as well as more accurately reflect performance. All simulations in the current study were held within a short period of time and with similar scenarios, thereby facilitating analysis and comparison between participating hospitals. While providing potentially vital information concerning HICGs’ response, the generalizability of the results may be questionable due to the relatively low number of simulations. However, this study, when added to the literature with similar results, strengthens the likelihood that these results may be transferable in similar settings. The wording of some of the indicators from the original tool have been adjusted for grammar [6].

## Conclusion

To our knowledge, this is the first study identifying specific decision-making indicators that are directly associated to overall performance of the hospital incident command group. There is a significant correlation between decision-making skills and staff procedural skills. Hospital incident command groups’ proactive decision-making abilities tended to be less developed than reactive decision-making abilities. Proactive decision-making skills are correlated to staff procedure skills and may be a predictive factor for overall hospital incident command performance. A lack of proactive decision-making ability may hamper efforts to mitigate the effects of a major incident.

While the results of this study provide important steps in understanding disaster preparedness at the command level, further research utilizing other types of simulations are needed before conclusions of causation and needs for definitive educational interventions can be drawn.

## Data Availability

The datasets used and/or analyzed during the current study are available from the corresponding author on reasonable request.

## Abbreviations

ANOVA: Analysis of variance
DiMI: Disaster Management Indicator tool
HICG: Hospital incident command group
MI: Major incident
